# Working memory is updated by reallocation of resources from obsolete to new items

**DOI:** 10.3758/s13414-022-02584-2

**Published:** 2022-10-17

**Authors:** Robert Taylor, Ivan Tomić, David Aagten-Murphy, Paul M. Bays

**Affiliations:** 1 University of Cambridge, Department of Psychology, Cambridge, UK; 2 University of Zagreb, Department of Psychology, Zagreb, CRO

**Keywords:** Visual working memory, short term memory, memory updating, resource reallocation, intrusion error

## Abstract

Visual working memory (VWM) resources are limited, placing constraints on how much visual information can be simultaneously retained. During visually-guided activity, stored information can quickly become outdated, so updating mechanisms are needed to ensure the contents of memory remain relevant to current task goals. In particular, successful deallocation of resources from items that become obsolete is likely to be critical for maintaining the precision of those representations still in memory. The experiments in this study involved presenting two memory arrays of coloured disks in sequence. The appearance of the second array was a cue to replace, rehearse, or add a new colour to the colours in memory. We predicted that successful resource reallocation should result in comparable recall precision when an item was replaced or rehearsed, owing to the removal of pre-replacement features. In contrast, a failure to update WM should lead to comparable precision with a condition in which a new colour was added to memory. We identified a very small proportion (~ 5%) of trials in which participants incorrectly reported a feature from the first array in place of its replacement in the second, which we interpreted as a failure to incorporate the information from the second display into memory. Once these trials were discounted, precision estimates were consistent with complete redistribution of resources in the case of updating a single item. We conclude that working memory can be efficiently updated when previous information becomes obsolete, but that this is a demanding active process that occasionally fails.

Working memory is constrained in how much information can be actively maintained at any given time, which in combination with its central role in supporting cognition means that memory contents are continually in flux. Consider the task of keeping a shopping list in memory while going to a store, then receiving a call to buy apple juice instead of orange juice: this requires discarding an irrelevant shopping item and reallocating freed memory capacity to the new item. Updating a spatial memory representation of nearby cars while driving is another example, although one with less trivial consequences of updating failure. It has been proposed that solving such tasks requires updating mechanisms to ensure memory resources are reorganized effectively and the contents of memory remain relevant to current task goals (e.g., Oberauer, 2001, 2018). The efficiency of these mechanisms may be crucial in dynamic environments where information can quickly become obsolete.

In human vision, the limited capacity of working memory has been observed as a decline in recall fidelity as the number of items in memory increases (Bays & Husain, 2008; Fougnie, Suchow, & Alvarez, 2012; Palmer, 1990; Schneegans, Taylor, & Bays, 2020; van den Berg, Shin, Chou, George, & Ma, 2012; Zhang & Luck, 2008). Successful deallocation of resources from obsolete items is therefore likely to be critical for maintaining the precision of task-relevant representations, while storage of new information requires resources to be reorganized. In retrospective cuing paradigms (Griffin & Nobre, 2003; Landman, Spekreijse, & Lamme, 2003; Maxcey-Richard & Hollingworth, 2013; Pertzov, Bays, Joseph, & Husain, 2013; Souza, Rerko, Lin, & Oberauer, 2014), an informative spatial cue presented during the delay interval directs attention toward an item that is no longer visible. Despite the observer only having access to the memory representation of the cued item, the retro-cue nonetheless improves recall of that item relative to non-cued items. The exact mechanisms responsible for the retro-cue benefit are debated (Souza & Oberauer, 2016). One recent study captured the effects of retro-cueing as an elevated amplitude of activity for the cued item relative to non-cued items in a neural population representation (Bays & Taylor, 2018). The increased strength of signal may protect the cued item representation in multiple ways: against passive loss of precision over time due to diffusion of stored values (Schneegans & Bays, 2018); against confusion with other items in memory (Oberauer & Lin, 2017; Schneegans & Bays, 2017); and against disruption by subsequent visual input (Souza & Oberauer, 2016; Tabi, Husain, & Manohar, 2019).

Often, changes in the environment, e.g., when visiting a store or driving a car, call for a rapid erasure of outdated information. Such requirements may not be well served by passive forms of information loss but instead require active processes that selectively remove irrelevant representations currently held in memory (e.g., Oberauer, 2001, 2018). This rapid erasure of outdated information subsequently frees up memory resources for the remaining task-relevant items (Souza, Rerko, & Oberauer, 2014) and has been shown to attenuate the effect of load on memory (Souza, Rerko, Lin, & Oberauer, 2014). In addition, several studies have demonstrated that individuals can simply *forget* information they no longer need when cued to do so (Williams & Woodman, 2012), to the point that any information pertaining to the removed items may become completely irretrievable (Williams, Hong, Kang, Carlisle, and Woodman 2013; see Lewis-Peacock, Drysdale, Oberauer, and Postle 2011, and Stokes 2015 for alternative accounts). These findings motivated the removal hypothesis of the retro-cue effect, which proposes that uncued items are simply removed from memory when a highly predictive retro-cue indicates the to-be-tested item (Souza & Oberauer, 2016).

The removal process itself is closely related to - and indeed has been proposed as a component process of - a more general cognitive mechanism referred to as *memory updating* (Ecker, Oberauer, & Lewandowsky, 2014; Kessler & Meiran, 2006, 2008). Updating tasks typically require an observer to replace existing memory representations when new information becomes available. In effect, items at particular spatial, or temporal, locations are overwritten and the observer must keep track of only the most recently presented information. In practice, this requires an obsolete item to be removed (i.e., resources are deallocated from it), followed by encoding of a new item (i.e., resources are allocated to it).

Ecker, Lewandowsky, and Oberauer (2014) demonstrated that a primary predictor of successful memory updating is the ability to efficiently remove outdated information. Intuitively, complete removal of obsolete items ought to facilitate the successful encoding of new information to the same contextual locations. Nevertheless, Ecker and colleagues argued that removal of outdated items is not a particularly easy task in contrast to studies that have claimed information can be wilfully discarded from memory (e.g., Williams et al., 2013; Williams & Woodman, 2012). Notably, most research on updating has used letter and digit stimuli within recognition-based paradigms. Accordingly, the precision of working memory has not been measured in these tasks, raising the possibility that items may have been degraded in fidelity rather than fully removed.

Here we investigated how efficiently resources can be deallocated from items that are no longer required, using a modified analogue recall task (Prinzmetal, Amiri, Allen, & Edwards, 1998; Wilken & Ma, 2004). Our working hypothesis was that successful deallocation of resources from an irrelevant item would mean it no longer counted towards the “effective” set size determining precision for the remaining items. Specifically, successful updating was expected to result in an enhancement of memory precision compared to a condition where the amount of information that had to be stored in memory was equal to the total number of presented relevant and irrelevant objects. Conversely, a failure to fully withdraw resources was expected to be seen as a precision cost to other items compared to that ideal. In particular, following successful object removal, the performance ought to be comparable to the precision achieved with a set size equal to the number of relevant items only.

## Experiment 1

In our first experiment we examined how well participants could update the contents of VWM when presented with a single additional piece of information. In particular, we were interested in whether resources encoding a formerly task-relevant item could be completely reallocated to a new item. In this and subsequent experiments, all updated (i.e., replaced) features became obsolete and were never tested.

### Method

#### Participants

A total of 12 participants (7 females, 5 males; aged 21−45 years, M = 26.4, SD = 6.2) took part in the study having provided informed consent in accordance with the Declaration of Helsinki. Participants all had normal or corrected-to-normal visual acuity and reported having normal colour vision.

#### Stimuli & Apparatus

Stimuli were presented on an LCD monitor (45 cm × 28 cm) with a refresh rate of 60 Hz. Participants were positioned 60 cm from the screen with their head supported by a chin and forehead rest. Eye position was monitored online at 1000 Hz using an infra-red eye tracker (Eyelink 1000, SR Research). To establish fixation we presented a central white fixation point (.25° radius) against a grey background. Study arrays consisted of coloured discs (1° radius) that were randomly located at one of four equidistant points around the circumference of an imaginary circle (6° radius) centred on the fixation point, with a rotational offset chosen randomly on each trial. Disc colour was determined by randomly sampling from a colour wheel, defined by a circle in CIELAB space with constant luminance (L*= 50), centre *a* * = *b* * = 20, and radius of 60. Test arrays consisted of a circular white annulus (1° radius) acting as a memory cue to indicate the location of the item to be recalled, and a colour wheel (3° radius) centred on the fixation point.

#### Procedure

**Figure 1:**
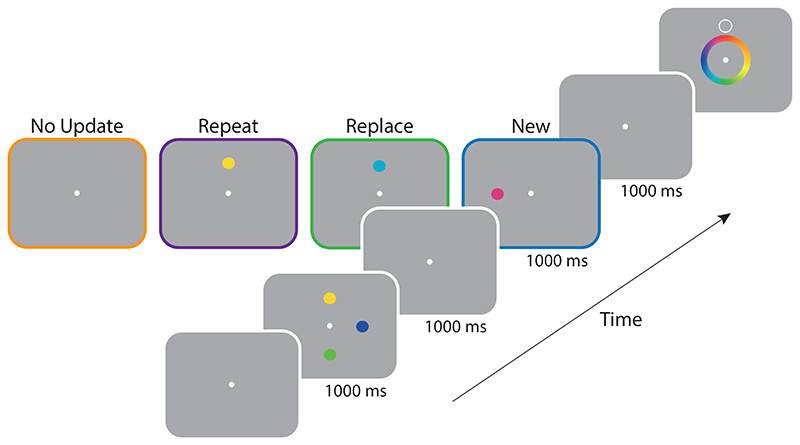
Procedure of Experiment 1. *Note.* After encoding an initial set of three coloured items, observers were subsequently shown one of four types of array: a *No Update* array (orange outline) contained no feature information; a *Repeat* array (purple outline) contained a single repeated item (here, the yellow disc); a *Replace* array (green outline) contained a new colour replacing the one previously shown at the same location (here, light blue replaces yellow); a *New* array contained a new colour at a previously unoccupied location (here, the pink disc). After a delay, a spatial cue was shown and the participant reported the corresponding colour in memory by selection from a colour wheel.

Following eye-tracker calibration, trials commenced with the presentation of a fixation point. Once a stable fixation had been established within 2° of the fixation point, the first study array was presented (1000 ms). This array always contained an initial set of three coloured discs. Participants were instructed to commit each of the three items to memory. A blank retention interval then followed (1000 ms), after which participants were shown one of four types of study arrays (1000 ms; see Figure 1).

In the *No Update* condition the second array remained blank and only required participants to maintain the original three items in memory (Figure 1, orange outline). Subsequently, a target item was selected at random from the first sample array. The purpose of introducing this condition was to ensure that observers would encode first-array colours throughout the experiment. Participants each completed a total of 54 *No Update* trials.

In the *Repeat* condition, one randomly chosen item was selected from the first array and presented again at the same location (Figure 1, purple outline). Although the second presentation of the item was physically identical to the first, we could not assume it coincided exactly with the colour in memory, so participants were instructed on *Repeat* trials to remember the most recently presented colour of the repeated item, while still maintaining memories of each non-repeated item. Participants completed a total of 72 *Repeat* trials, with each location cued equally often (i.e. 24 trials for the repeated item and 24 for each of the non-repeated items).

In the *Replace* condition, a disc with a new randomly chosen colour, which we term the *post-replacement stimulus*, was presented at the same location as one of the original three items (Figure 1, green outline). Participants were again instructed to remember only the most recently presented colour and told that they would not be tested on the original colour, which we define as the *pre-replacement stimulus*. As a result, the *Replace* condition required the participant to update a single item in memory while continuing to maintain representations of the other two items from the first array. Participants completed a total of 72 *Replace* trials, with each location cued equally often (i.e. 24 trials for the replaced item and 24 for each of the non-replaced items).

Finally, in the *New* condition, a disc with a randomly chosen colour was presented at a new array location that was unoccupied in the first array (Figure 1, blue outline). On these trials participants were told to remember the fourth colour in addition to the original three items. Participants completed a total of 72 *New* trials, with each item cued equally often (i.e. 18 trials for the item in the second array and 18 for each of the three items in the first array).

In total, each participant completed 270 trials in a single one hour session. To avoid any uncertainty over trial types, we tested the potentially confusable *Replace* and *Repeat* conditions in two separate blocks, giving participants instructions applicable for each. One block randomly interleaved *No Update* (27 trials), *Replace* (72 trials), and *New* (72 trials); the other block interleaved *No Update* (27 trials) and *Repeat* (72 trials). Block order was counterbalanced across participants. Eye tracking was used to monitor fixation. If gaze position deviated by more than 2 dva before onset of the response cue, a message appeared on the screen, and the trial was aborted and restarted with newly randomized colours later in the same block.

### Analysis

We measured recall error across each condition as the angular deviation between the reported and target colours on the colour wheel. The dispersion of recall errors, measured as circular standard deviation, was used to estimate how precisely items were retrieved from memory. We used Bayesian statistics for evaluating the evidence for and against our hypotheses, implemented in JASP (JASP Team, 2020) using the default Jeffreys-Zellner-Siow prior on effect sizes (Liang, Paulo, Molina, Clyde, & Berger, 2008). The Bayes factor compares the predictive adequacy of two competing hypotheses (e.g., alternative and null) and quantifies the change in belief that the data bring about for the hypotheses under consideration (Wagenmakers et al., 2018). For example, BF_10_ = 5 indicates that the data is five times more likely to occur under the alternative than the null hypothesis. Evidence for the null hypothesis is indicated by BF_10_ < 1, in which case the strength of evidence is indicated by 1/BF_10_. When presenting results from Bayesian ANOVAs we report the overall evidence for an effect. This is derived via Bayesian model averaging which averages over all candidate models that contain the effect of interest.

**Figure 2:**
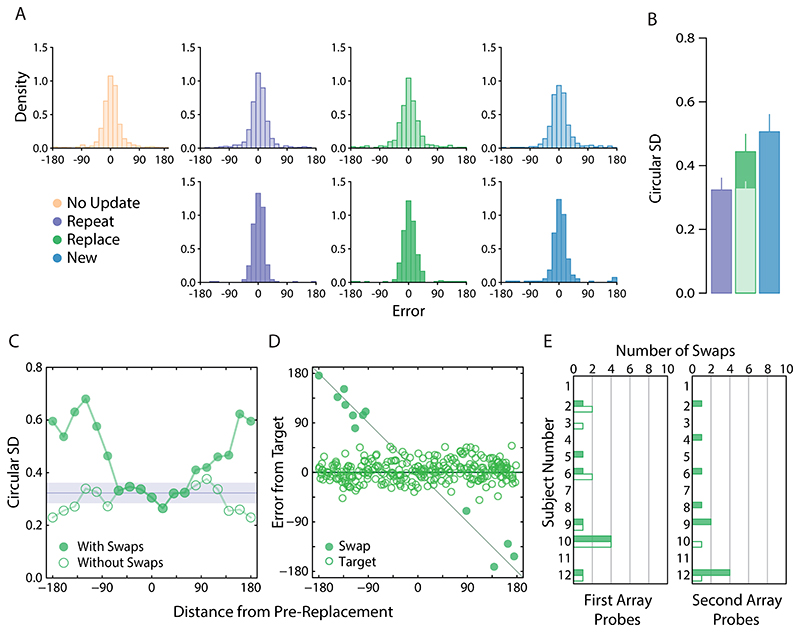
Experiment 1 results. *Note.* **a)** Participant pooled error distributions. Lighter histograms reflect recall error for first array items, darker histograms for second array items. **b)** Corresponding circular standard deviation for second array probes only. Bars denote participant-averaged performance across each condition. The lighter green bar indicates group performance following removal of pre-replacement intrusions (as opposed to panel a)). Error bars indicate ± 1 SEM. **c)** Circular SD as a function of distance between features values presented at the replaced location. **d)** Trial-by-trial response errors for recall of replacement item feature, plotted as a function of distance between feature values presented at replacement location. Filled circles indicate all swap errors, i.e., intrusion errors and conventional non-target responses. **e)** Number of non-target reports in the *Replace* condition. Filled bars denote the number of pre-replacement intrusions. Unfilled bars are the number of conventional non-target reports.

We examined effects of the similarity between pre- and post-replacement items (in the *Replace* condition) by calculating recall variability as a function of the angular difference between the colours on the colour wheel. We did this by first pooling responses across observers (Figure 2A, dark green distribution, bottom panel) and then calculating the circular SD for 19 equally spaced colour distances, based on overlapping bins with widths of 50°.

We additionally fit the three component mixture model to the response distributions from each condition (Bays, Catalao, & Husain, 2009; code available at https://bayslab.com/toolbox/). This model assumes a probabilistic mixture of responses distributed (a) around the target colour, (b) around each of the other, non-target colours presented on a trial, and (c) uniformly on the colour wheel. Owing to the small number of trials in individual conditions at the participant level, we fit the model to pooled participant data to obtain more reliable parameter estimates. The mixture model estimates are based on maximum likelihood principles, meaning that this method requires a sufficiently large number of observations to find the true parameter values with arbitrary precision (i.e., consistency property). In particular, simulations conducted using the mixture model have shown that the obtained number of trials at the participant level in Experiment 1 (i.e., <30 trials) is unlikely to provide reliable parameter estimates (https://bayslab.com/toolbox/), making it necessary to estimate parameters using pooled data.

To be sure of detecting all possible intrusion (swap) errors, every colour presented on a trial was entered into the mixture model, either as the target or as a non-target. On *Replace* trials, if the replaced item was cued (i.e., indicated for recall) then the post-replacement colour was the target and the pre-replacement colour was entered as a non-target, while if one of the other items was cued both pre- and post-replacement colours were entered as non-targets. In the *New* condition, the cued item was the target and the other three colours were entered as non-targets, irrespective of the array in which they were presented.

To distinguish between different kinds of intrusion error, we first used the trial-by-trial probability weights from the global mixture fit to classify as *swap trials* all trials where the posterior probability for the non-target component exceeded target and uniform components. Having identified these trials, we next inferred which non-target colour was most likely to have been reported in place of the target in each trial by examining the absolute distances between the reported colour and each non-target colour. Of particular importance, we identified a *pre-replacement intrusion* as any swap trial on which the pre-replacement colour was closest to the reported colour.

### Results

The updating task of Experiment 1 was designed to yield clear quantitative predictions for the *Replace* condition. If the obsolete (pre-replacement) colour was efficiently removed from memory, then the allocation of resources to items should be the same as in the *Repeat* condition, with an effective set size of three. On the other hand, if the obsolete colour could not be removed and continued to occupy resources in the same way as the other items, the allocation should be the same as in the *New* condition, i.e. an effective set size of four. Note that, while both the *No Update* and *Repeat* conditions had an effective set size of three, the *Repeat* condition was designed to match *Replace* also in the delays between stimulus presentation and cue.

Our main results are plotted in Figure 2, which shows the pooled response distribution for each condition (Figure 2A; top row first array items; bottom row second array items), along with the corresponding circular SD estimates for the second array probes only (Figure 2B). To address our central hypothesis we began by comparing recall precision between the second array probe conditions, i.e. when the new item (*New* condition), repeated item (*Repeat* condition) or post-replacement item (*Replace* condition) was indicated for report. As the experiment was carefully designed to contrast the precision of second-array probes, precision differences for first-array probes are consistent with multiple hypotheses, some of them unrelated to memory updating. Therefore, their analysis is provided in Supplementary Materials.

For this initial analysis, we found that recall variability was greater for new items (dark blue bars) than for repeated items (dark purple bars; *δ* = -1.20, 95% CI :[-2.15, -0.34], BF_10_ = 10.07), but was similar in magnitude when compared to post-replacement items (dark green bars; δ = -.27, 95% CI :[-1.00, 0.38], BF_10_ =.42). However, no conclusive difference could be detected between post-replacement and repeated items (δ = -.53, 95% CI :[-1.34, 0.18], BF_10_ =.94).

Because pre- and post-replacement colours were chosen independently at random, on some proportion of *Replace* trials the replacement colour could have been very similar to the colour it replaced. To examine the effect of this similarity we plotted recall variability as a function of the angular difference between colours of pre- and post-replacement items. The result is plotted in Figure 2C (filled circles). Group performance in the *Repeat* condition is shown for comparison (purple region).

Values close to zero on the *x* -axis imply a high degree of colour similarity between the first- and second-array items presented at the replacement location. Perceptually, these instances will mimic a *Repeat* trial and, indeed, recall variability is very similar between the two conditions when colour similarity is high. However, we observed a substantial increase in variability when pre- and post-replacement items became more dissimilar.

To investigate further, we plotted trial-by-trial responses for each observer as a function of colour similarity between pre- and post-replacement items (Figure 2D). This revealed a very clear explanation for why variability was greater for increasingly dissimilar colours. Though the majority of responses were clustered around the (correct) post-replacement colour, we observed a handful of errors that were displaced along the negative diagonal, consistent with observers erroneously reporting the pre-replacement colour. This would tend to inflate variability estimates when pooled with other trials.

Mixture model fits further confirmed this interpretation. Our analysis identified a very small number of pre-replacement intrusions (only 10 in total; 3.47% of *Replace* trials on which the post-replacement item was probed), each coinciding with one of the data points falling along the negative diagonal (indicated by filled circles). We also noted a similar proportion of non-target reports on *Replace* trials where one of the non-replacement items was probed (3.47% of trials).

**Figure 3:**
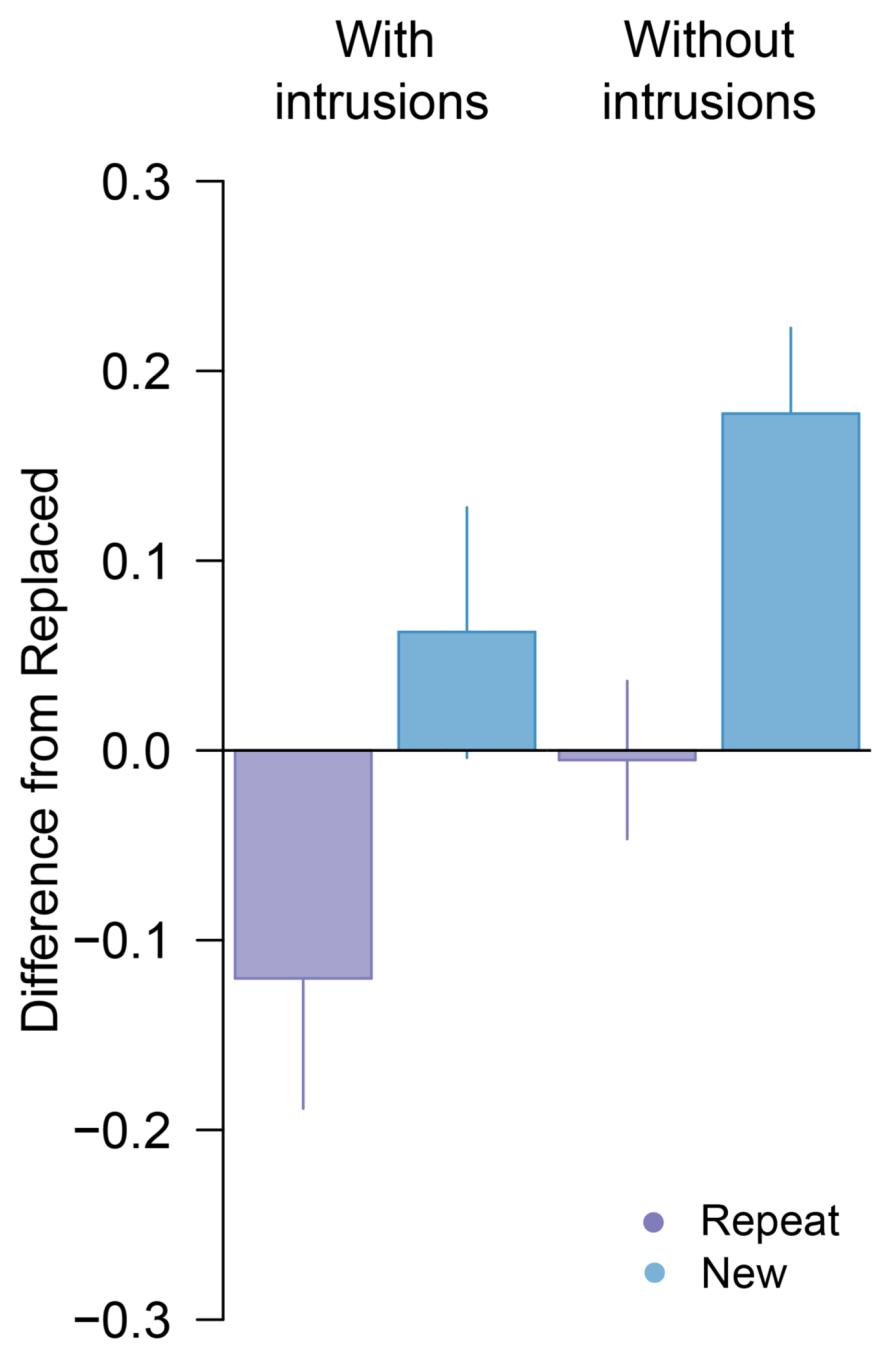
Difference in circular standard deviation for second array targets in Exp. 1. *Note.* Difference between the *Replace* and *Repeat* (purple) or *New* (blue) condition, before (left) and after (right) removing intrusion errors. Positive values indicate performance was better (lower variability) in the *Replace* condition. Error bars indicate ±1 SEM.

Critically, when we removed the 10 trials identified as pre-replacement intrusions from analysis, we found post-replacement recall variability to be approximately invariant with colour distance (Figure 2C, unfilled circles). There was now strong evidence for a difference in precision between post-replacement (Figure 2B, lighter shaded bar) and new items (Figure 3; δ = -1.36, 95% CI :[-2.33, -0.31], BF_10_ = 19.5), and no consistent difference from recall of repeated items (δ = -0.04, 95% CI :[-0.73, 0.63], BF_10_ = 0.29).

### Discussion

In the first experiment we successfully demonstrated that working memory resources can be efficiently reallocated from obsolete to relevant information. Critically, this was found only after discounting a very small proportion of trials on which observers failed to reallocate resources and reported the to-be-replaced colour. Although recall performance was overall worse on *Replace* than *Repeat* trials, an inspection of the trial-by-trial data revealed that this decrement was fully accounted for by a very small number of systematic failures where individuals mistakenly reported a pre-replacement stimulus feature. When influence of those trials was removed, precision estimates were aligned with performance in the *Repeat* condition where only three items were encoded into memory.

The removal of intrusion errors should not be considered a mere data cleaning procedure. Instead, there are two main points to be taken regarding those errors. First, the identification of intrusion errors allowed us to uncover a specific way in which memory updating fails. Second, despite their comparative infrequency, these intrusions had a profound influence on our measure of recall precision. Following their removal, we were able to assess how efficient resource allocation was on the remaining majority of trials.

Our results extend and clarify those of Kessler et al. (2015), who reported advantageous change detection accuracy for repeated relative to updated items. Prior to the removal of intrusion errors, the results of our initial analysis revealed a similar pattern of data to that observed by Kessler et al. (2015). This suggests that the accuracy cost for updated items observed in that study may correspond to occasional intrusions of a pre-replacement item, as we observed.

The absence of difference between *Replace* and *Repeat* provides only partial evidence of successful resource reallocation. An equally important piece of evidence consists of demonstrating that the cost of updating is smaller than committing the additional item to memory. Such a cost would occur if the obsolete information could not be removed from memory and continued to occupy resources, affecting the precision of relevant objects. Although our initial analysis was ambiguous regarding this cost, following the removal of intrusion errors we found strong evidence that the precision cost of updating an existing item was smaller than storing an additional item. Together, comparisons of *Replace* with *New* and *Replace* with *Repeat* provided converging evidence that on the large majority (>95%) of trials, participants successfully reallocated resources from the obsolete item to its replacement.

It remains unclear exactly why individuals occasionally failed to update their memories. One simple explanation is that these errors reflect momentary confusion about the task instructions: the subject may have temporarily forgotten which items they were supposed to remember, or mistaken a *Replace* trial for a *New* trial. Another possibility is that replacing only a subset of all encoded items is a cognitively more challenging operation than simply replacing all items. For example, Kessler and Meiran (2008) argued that “partial-set updating”, i.e. changes made to a subset of items in memory, requires a complex series of steps involving the decoupling and substitution of individual item features within each encoding context.

In comparison, “whole-set updating” − where all items in memory are updated − is arguably a much simpler process. In this case the entire contents of memory can be discarded and completely new information encoded in its place. Because whole-set updating does not require removal to proceed in an item-wise fashion it is claimed to occur more quickly (Kessler & Meiran, 2008). It is conceivable, then, that the rare persistence of pre-replacement features in memory might be a consequence of individual updating. To test this hypothesis, the next exp eriment required observers to update the entire contents of memory on some trials.

## Experiment 2

**Figure 4:**
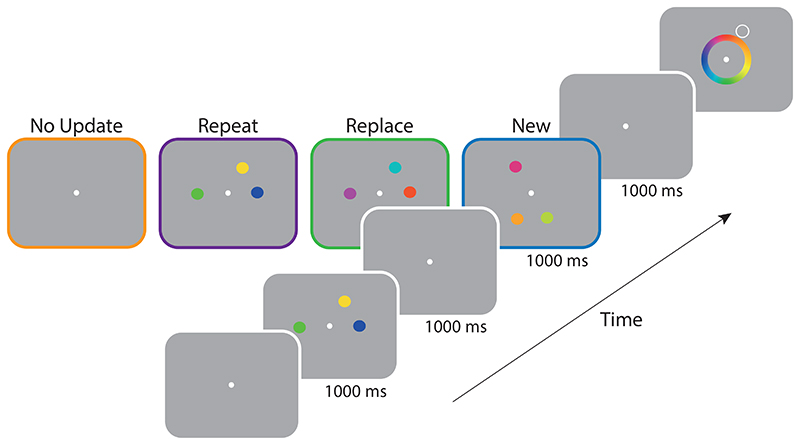
Procedure of Experiment 2. *Note.* After encoding an initial set of three coloured items, observers were subsequently shown one of four types of array: a *No Update* array contained no feature information; a *Repeat* array contained three repeated items; a *Replace* array contained three new colours replacing those previously shown at the same locations; a *New* array contained three colours at previously unoccupied locations. After a delay, a spatial cue was shown and the participant reported the corresponding colour in memory by selection from a colour wheel.

To investigate whole-set updating of working memory, second arrays in Experiment 2 contained three items instead of just one (Figure 4). In other respects the design was very similar to Exp. 1.

### Method

#### Participants

A total of 12 new participants (12 females; aged 21–30 years, M = 25.1, SD = 3.4) took part in the study having provided informed consent in accordance with the Declaration of Helsinki. Participants all had normal or corrected-to-normal visual acuity and reported having normal colour vision.

#### Procedure

Study arrays again consisted of coloured discs (Figure 4). The location of each coloured disc was chosen at random from six equidistant locations positioned on an imaginary circle and rotated by a random offset from trial to trial. The *No Update* condition (54 trials per participant) was identical to Exp. 1. In the *Repeat* condition (72 trials), all three items in the first array were presented again in the second array. In the *Replace* condition (72 trials), three new coloured discs were presented at the same locations as the original three items. In this condition participants were instructed to remember the most recently presented colours and that the original items would not be tested.

Finally, in the *New* condition (72 trials), three new coloured discs were presented at new array locations. On these trials participants needed to encode the new items while continuing to maintain the items in the first array. Targets were selected from either array with equal frequency. Participants competed a total of 270 trials in a single one hour session, with sessions blocked in the same way described for Experiment 1.

### Results

For our second experiment, the predictions are essentially the same as Exp. 1: if observers can efficiently replace obsolete items, then only three items should be represented in memory in the *Replace* condition, making the effective set size the same as in the *Repeat* condition. If the obsolete items are not removed, the effective set size is six, matching the *New* condition.

**Figure 5:**
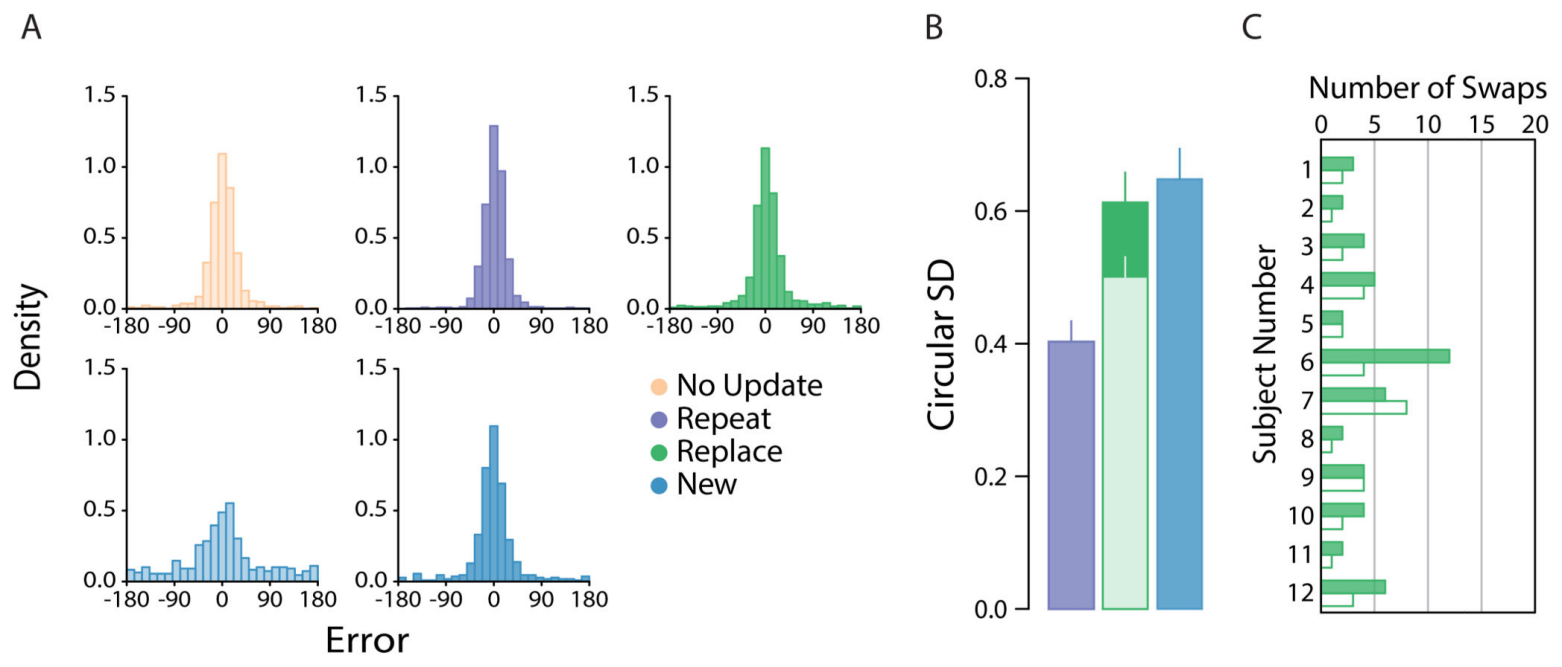
Experiment 2 results. *Note.* **a)** Participant pooled error distributions. Lighter histograms reflect recall error for first array items (No *Update* and *New* conditions only), darker histograms for second array items. **b)** Corresponding circular standard deviations for second array probes only. Bars denote participant-averaged performance in each condition. The lighter green bar indicates performance following removal of pre-replacement intrusions. **c)** Number of non-target reports. Filled bars denote pre-replacement intrusions, unfilled bars conventional non-target reports.

The results are plotted in Figure 5. Bayesian repeated measures *t*-tests showed strong evidence for better performance for repeated items than either post-replacement (δ = -1, 95% CI :[-1.77, -0.29], BF_10_ = 21.94), or new items (δ = -1.6, 95% CI :[-2.58, -0.69], BF_10_ = 422). When comparing performance between post-replacement and new items, the data moderately supported the null hypothesis of no difference (δ = -0.13, 95% CI :[-0.65, 0.38], BF10 = 0.33). Accordingly, preliminary analyses again suggested that observers were imperfectly removing pre-replacement features.

We next considered the extent to which pre-replacement intrusions might have influenced this result. Despite asking observers to globally update their memory representations, our mixture analysis indicated that pre-replacement intrusions occurred at a somewhat higher rate (6.02% of trials in the *Replace* condition) to the previous experiment. In addition, in the *Replace* condition we also observed a number of within-display swaps (3.94%), i.e., reporting a non-target presented in the second array.

**Figure 6:**
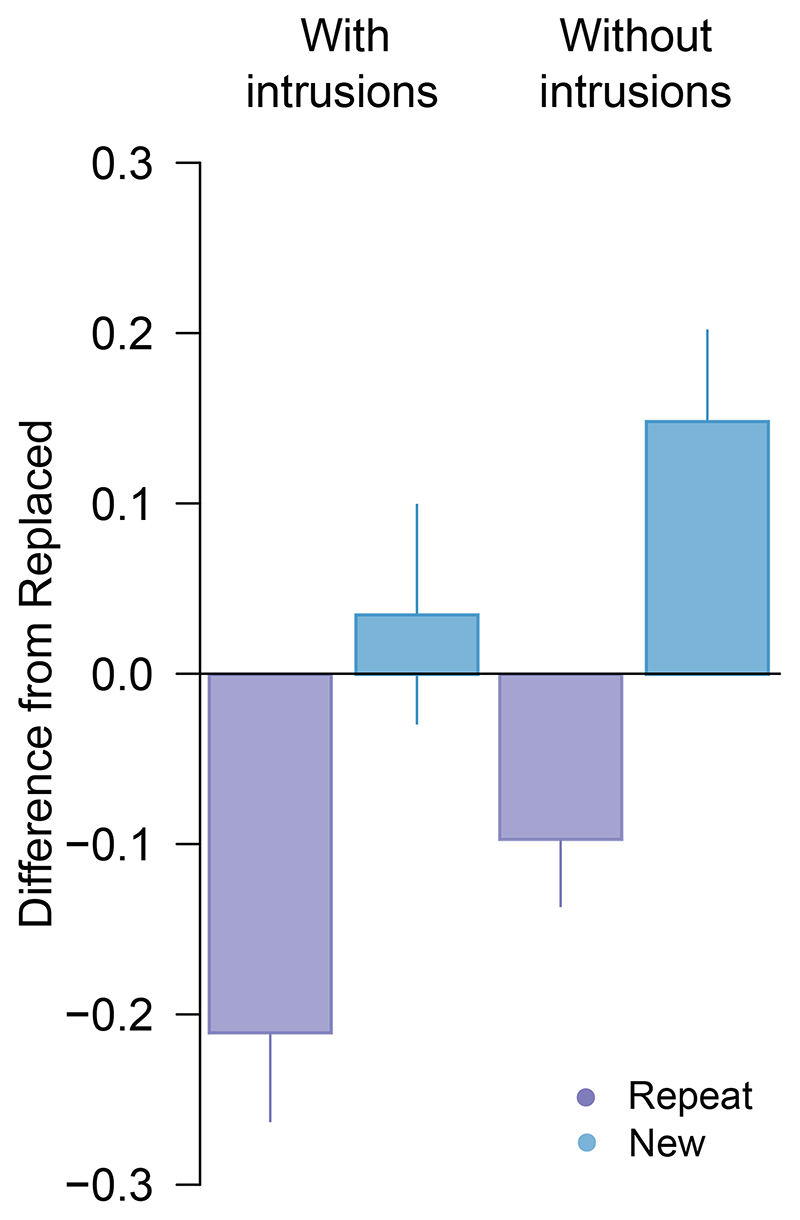
Difference in circular standard deviation for second array targets in Exp. 2. *Note.* Difference between the *Replace* and *Repeat* (purple) or *New* (blue) condition, before (left) and after (right) removing intrusion errors. Positive values indicate performance was better (lower variability) in the *Replace* condition. Error bars indicate ±1 SEM.

Subsequent removal of pre-replacement intrusions produced some reduction in the variability estimate for post-replacement features (Figure 5, lighter green bar). Critically, the corrected estimate yielded moderate evidence for better recall of post-replacement than new items (δ = -0.67, 95% CI :[-1.33, -0.07], BF10 = 3.56) as well as weak evidence for worse recall compared to repeated items (δ = -0.59, 95% CI :[-1.23, -0.01], BF_10_ = 2.32) (Figure 6).

### Discussion

The primary purpose of our second experiment was to examine the possibility that partial-set updating was responsible for updating failures in Exp. 1. In particular, ongoing maintenance of part of the memory content might disrupt a complex sequence of actions required for updating a single item, resulting in the occasional updating failures. To address this we instead asked observers to globally update all items in memory by presenting three entirely new items in the second stimulus array. Specifically, to avoid any deleterious effects of simultaneous memory maintenance on resource reallocation, we asked observers to forget old items and encode new items all at once. We nevertheless observed a similar rate of pre-replacement intrusions between the two experiments. Whole-set updating, then, had no appreciable effect upon how frequently pre-replacement intrusions occurred. Despite the low incidence of intrusions, they had a similarly deleterious effect on variability estimates. Indeed, when removed from the *Replace* condition data, the estimated variability was again lower than the *New* condition, consistent with successful deallocation of resources from obsolete items on the large majority of trials, although the fact that variability did not fall as low as the *Repeat* condition suggests a complete reallocation of resources from all three pre-replacement items may not have been achieved. Such partial resource reallocation is in contrast with findings from Exp. 1 where memory precision in *Replace* and *Repeat* conditions coincided.

The comparable rates of pre-replacement intrusion in Experiments 1 and 2 suggest that whole-set updating did not benefit the removal of outdated information, as previously suggested Kessler and Meiran (2008). This, along with observed partial resource reallocation, raises the possibility that resources can only be deallocated item-by-item. Theoretical work by Ecker, Oberauer, and Lewandowsky (2014) showed that updating can be explained by a model that must first decouple item-context bindings in an item-wise fashion, regardless of number, before any items can be removed.

It has further been argued that item-wise removal requires both time and effort (Fawcett & Taylor, 2008; Oberauer, 2001) to complete. Whereas it takes 50−100 ms to encode a single item into memory (Bays, Gorgoraptis, Wee, Marshall, & Husain, 2011; Vogel, Woodman, & Luck, 2006), removal of the same amount of information is a comparatively slower process, estimated at 500−600 ms (Ecker, Lewandowsky, & Oberauer, 2014). Earlier work using word lists has also provided estimates upward of two seconds for the complete removal of three items (Oberauer, 2001).

If a similar amount of time is required for removal of three visual features from working memory, this could explain why we saw incomplete reallocation of resources in Exp. 2. We examined this possibility in our third experiment.

## Experiment 3

In this experiment we examined whether additional time would allow for more complete removal of obsolete items from memory. We used the same basic updating procedure as before, but introduced a temporal manipulation intended to provide more time to remove obsolete working memory contents following the presentation of the second stimulus array. If the removal of irrelevant items is a time-consuming process, as previously suggested (e.g., Oberauer, 2001), allowing more time should increase the frequency with which removal was successful and consequently reduce the frequency of intrusion errors. As the main focus of this experiment was on intrusion errors, we used only the *Replace* and *New* conditions.

### Method

#### Participants

A total of 12 new participants (7 females, 5 males; aged 19−39 years, M = 27, SD = 5.4) took part in the study having provided informed consent in accordance with the Declaration of Helsinki. Participants all had normal or corrected-to-normal visual acuity and reported having normal colour vision. We identified two participants, via the mixture model analysis, that had misunderstood the task. These individuals were found to have reported the pre-replacement instead of the post-replacement colour on almost all trials in the *Replace* condition. While this potentially indicates an inability to update WM contents, we believe a more likely explanation is that these observers misunderstood the task, since the remaining observers across all experiments showed very low or zero rate of intrusions. We therefore excluded these participants from further analysis. Keeping them in the sample would have led to the exclusion of the majority of their trials as intrusion errors and not meaningfully changed the outcome of the analysis.

#### Procedure

The experiment consisted only of *Replace* and *New* conditions, which were identical to those in Exp. 2 (Figure 4), except for a variable delay interval followed the offset of the second array of 1, 2, or 4 seconds. Condition and delay interval were randomly interleaved and participants completed two blocks of 144 trials in a single one-hour session.

### Results

**Figure 7:**
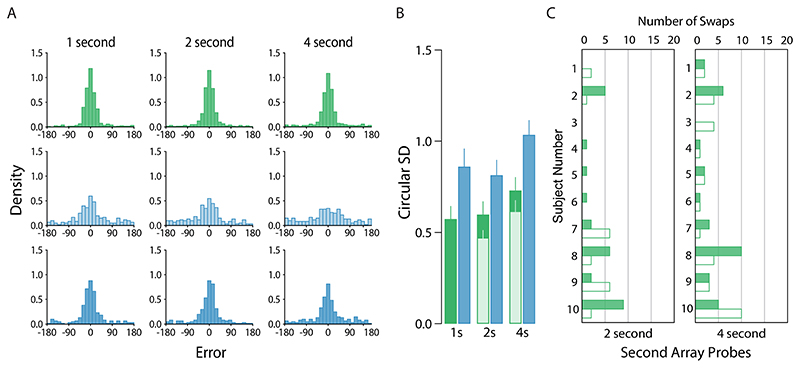
Experiment 3 results. *Note.* **a)** Participant pooled error distributions. Lighter histograms show recall error for first array items (New condition only), darker histograms for second array items. **b)** Corresponding circular standard deviations for second-array probes only. Bars denote participant-averaged performance across each condition. The lighter green bars indicate performance following removal of pre-replacement intrusions. Note that no lighter bar is presented for the 1s condition because no non-target responses were identified. **c)** Number of non-target reports. Filled bars denote pre-replacement intrusions, unfilled bars conventional non-target reports.

The main results from Experiment 3 are plotted in Figure 7. In contrast to our previous two experiments, we found strong evidence that observers recalled post-replacement items more precisely then new items, even before accounting for pre-replacement intrusions (2x3 Bayesian repeated measures ANOVA: BF_*indusion*_ = 7.13 × 10^5^). We also found strong evidence for an effect of delay (BF_*indusion*_ = 49.4), though the effect appeared to be driven by poorer recall at the longest delay interval (1 second vs. 2 second: BF_10_ = 0.24; 2 second vs. 4 second: BF_10_ = 58.5). We found no evidence for an interaction between condition and delay BF_*indusion*_ = 0.27).

Our mixture analysis did not detect any pre-replacement intrusions in the shortest delay interval. However, intrusion rates in the *Replace* condition were comparable with previous experiments for both the two-second (5.63%) and four-second (6.88%) condition. The rates of other non-target reports in the same condition were also low but appeared to increase with delay interval, and we found 0%, 3.96%, and 6.67% of within-array swap errors for one-second, two-second, and four-second conditions, respectively.

Removal of pre-replacement intrusions reduced estimated SD in both the 2 and 4 s delay condition (Figure 7B, lighter shaded bars) but the overall pattern of statistical results did not change (2x3 Bayesian repeated measures ANOVA: memory condition BF_*indusion*_ = 1.31 × 10^8^; delay BF_*indusion*_ = 13.27; interaction BF_*indusion*_ = 1.29).

### Discussion

Our third experiment investigated whether observers would benefit from having more time to remove items from working memory. We varied the delay interval between the offset of the second array and the probe array to examine this possibility. We found that recall of replacement items was consistently better compared to the recall of new items across all delays, even when the influence of pre-replacement intrusions was left uncorrected. Nevertheless, these results largely discount the conjecture that observers would benefit from more time to remove information from memory. At the longest delay intervals, pre-replacement intrusions continued to occur at a similar low rate to previous experiments. Once these intrusions were removed, variability in the *Replace* condition was not consistently influenced by delay interval.

## General Discussion

The present work explored whether observers could reallocate their visual working memory resources to accommodate new stimuli and remove obsolete information from memory. We presented one or more new stimuli at previously occupied locations to indicate to participants they should discard and replace information in memory corresponding to those locations. When comparing conditions that were matched in the timing and total number of items presented, the opportunity to replace items was expected to reduce the number of items maintained simultaneously in memory, resulting in more precise recall of the items remaining.

Across three experiments, our results indicated that individuals were able to update their working memories efficiently on most trials, reallocating all or most of the resources dedicated to obsolete items to store new information. Importantly, we also observed resource reallocation occasionally failing. Indeed, an important caveat is that the successful updating was revealed only after discounting a very small number of pre-replacement failures, i.e., trials where a participant was cued to report a replacement colour but reported the original colour instead. Despite their infrequency, because responses on these trials were uniformly distributed with respect to the target, their inclusion had a disproportionate effect on variability estimates that tended to obscure the benefit of successful replacement that occurred on the large majority of trials.

In order to evaluate the success of memory updating, we took advantage of the well-established set size effect on recall precision. Optimally efficient memory updating would result in an “effective set size” equal to the total number of relevant items after presentation of the second array. Conversely, a complete failure to discard obsolete objects would result in an effective set size equal to the sum of both relevant and irrelevant items. Therefore, a pattern of results indicating optimal memory updating would be signified by both a statistical equivalence in performance to a memory condition with the former set size and a statistical difference from a condition with the latter set size. While theoretically equally import ant, strong evidence as measured by the Bayes Factor for the former result could be harder to achieve in practice than for the latter result, given the asymmetry in how evidence for the null (i.e., absence of difference) and alternative (i.e., difference) accumulate (Keysers, Gazzola, & Wagenmakers, 2020; Stefan, Gronau, Schönbrodt, & Wagenmakers, 2019). Moreover, empirical patterns of recall precision might deviate from this desirable pattern, indicating only partial resource reallocation was achieved. While the results of Exp. 1 suggested complete memory removal is attainable, results of Exp. 2 were more ambiguous, suggesting updating may have been incomplete, either due to resources not being completely withdrawn from the obsolete object or not successfully allocated to the relevant object.

We observed considerable consistency in intrusion rates across our experiments, despite attempting to manipulate factors that could interfere with updating of memory. In our second experiment we examined whether global replacement of all items in memory was more efficient than replacing just one (Kessler & Meiran, 2008), while our third experiment examined whether updating would benefit from more time to unbind obsolete items from their encoding context (Ecker, Lewandowsky, & Oberauer, 2014; Ecker, Oberauer, & Lewandowsky, 2014). The relative invariance of intrusion rates across these experiments, and the similarity in recall variability once intrusions were removed, suggests that neither of these manipulations had a significant effect on updating efficiency.

The delay manipulation in Exp. 3 was intended to allow varying amounts of time for removal of obsolete information before memory was probed. The hypothesis that pre-replacement intrusions would become more infrequent with delay time was not supported, however one possible explanation is that the disengagement of memory resources from pre-replacement stimuli stopped once the second array disappeared, i.e. once there was no visible stimulus for them to be reallocated to. Although there would be no performance advantage to retaining pre-replacement stimuli on *Replace* trials (reporting them would be no better than guessing at random with respect to the instructed task) we cannot rule this account out based on our data. However, a recent study that tested whole-set updating of location information provides some evidence against this: Tabi et al. (2021) still observed intrusions of pre-replacement locations despite a much longer post-replacement stimulus duration (4 s) than in our study, suggesting prolonged exposure to replacement stimuli is not sufficient to achieve complete removal. Another possible approach would have been to cue items from the first array that were going to be replaced at varying intervals before the second array appeared (as in Ecker, Lewandowsky, & Oberauer, 2014), which would manipulate the time available for removal independently of encoding replacement information.

A simple explanation for the intrusion errors observed in these experiments is that they reflect transient failures of vigilance or attention to the stimuli during presentation of the second array. If an observer did not store the new information in the second array they would report a pre-replacement colour in response to the probe. Another possibility is that participants occasionally mistook a *Replace* trial for a *New* trial, and so intentionally retained the pre-replacement colours from the first array in addition to the post-replacement colours. However, we think this is unlikely: the unique locations used for stimuli on a trial were all widely spaced, so we doubt the location of a pre-replacement item in the first array would be recalled so inaccurately as to mistake its replacement at the same location in the second array for a new item at a different location. Even if that mistake were to be made, to result in a pre-replacement intrusion the participant would further have to match the location of the probe to the (putatively) misremembered pre-replacement location rather than to the post-replacement location to which it actually corresponded. We view this particular combination of individually improbable events as a less parsimonious explanation than occasional lapses in encoding the second array.

Whatever the cause of the rare intrusions, our results highlight the importance of identifying such contaminant responses, which can have a highly disproportionate influence on variability estimates. In the present case, failing to account for the possibility of pre-replacement intrusion would have led us to almost exactly opposite conclusions to the ones we have reached about the efficiency of updating. As we have emphasized previously (Taylor & Bays, 2018, 2020), mixture models can be a useful statistical tool to de-noise data even if mechanistic interpretations of the mixture components are uncertain.

Although a large body of literature demonstrates that people can deliberately erase memory content, understanding the mechanisms by which this occurs has been challenging. Results of studies conducted by Kessler and Meiran (2006, 2008) suggested that object removal involves “dismantling” or “unbinding” the old representations. This idea was more formally conceptualized and implemented in the SOB-CS model of working memory complex span task (Ecker, Lewandowsky, & Oberauer, 2014; Oberauer, Farrell, Jarrold, Pasiecznik, & Greaves, 2012), but the logic applies to other WM tasks (Lewis-Peacock, Kessler, & Oberauer, 2018). Here, active information removal is accomplished by breaking the association between the memory content (e.g., colour) and its context (e.g., spatial position of coloured disk). In particular, removing an object proceeds by cueing the object with its location and then unlearning the association between the object and its location. In SOB-CS this unbinding process is implemented as Hebbian unlearning: unlike Hebbian learning, which forms item-context binding, unlearning simply removes previously formed associations.

Previous studies have provided complementary neurophysiological evidence for the processes supporting memory updating, specifically the removal of items from WM and encoding of post-replacement items. Using multivariate decoding of EEG (LaRocque, Lewis-Peacock, Drysdale, Oberauer, & Postle, 2013) and fMRI activity patterns (Lewis-Peacock, Drysdale, Oberauer, & Postle, 2012) during WM maintenance, previous studies have found that presenting a cue indicating which item stored in memory is relevant for the task leads to an attenuation of decoder evidence for an uncued item relative to the cued item, consistent with removal of irrelevant information. More recently, this finding was extended by asking observers not only to remove memory content, but to replace it with new information (Kim, Smolker, Smith, Banich, & Lewis-Peacock, 2020). Similarly to previous studies, the item’s neural representation declined to baseline following the mid-trial instruction to replace that item with a new one. Furthermore the decline in decodability for the pre-replacement item was accompanied by an increase in classifier evidence for the post-replacement item. Together, these studies provide evidence for neural markers of WM content control mechanisms. Future research could aim to identify a neural signature of the occasional failures of resource reallocation that lead to intrusions of pre-replacement items.

Research on WM updating has important implications for understanding the etiology and symptoms of psychiatric disorders. In particular, it has been shown that WM updating deficits characterize many psychiatric disorders including depression (Levens & Gotlib, 2010; Meiran, Diamond, Toder, & Nemets, 2011), post-traumatic stress disorder (Moores et al., 2008; Weber et al., 2005), schizophrenia (Galletly, MacFarlane, & Clark, 2007; van Raalten, Ramsey, Jansma, Jager, & Kahn, 2008), and autism (Lieder et al., 2019). In those conditions, a failure to update WM with new information can result in intrusive thoughts and perseverative, maladaptive behavior. Better understanding the mechanisms of WM updating could therefore aid in designing more focused interventions specifically aimed at treating cognitive impairments in those psychiatric populations.

Taken together, our study provides evidence for efficient updating of working memory. Despite rare failures to update, our results clearly support the principle that observers can reallocate VWM resources from obsolete memoranda in order to maintain high precision representations of goal-relevant items. Future work could explore how these mechanisms operate in more naturalistic tasks and conditions requiring frequent updating of memory content.

## Supplementary Material

Supplementary Material

## Data Availability

Data and analysis code will be made publicly available upon acceptance of this manuscript. All data and code associated with this article can be found at https://doi.org/10.17863/CAM.88835.

## References

[R1] Bays PM, Catalao RFG, Husain M (2009). The precision of visual working memory is set by allocation of a shared resource. Journal of vision.

[R2] Bays PM, Gorgoraptis N, Wee N, Marshall L, Husain M (2011). Temporal dynamics of encoding, storage, and reallocation of visual working memory. Journal of Vision.

[R3] Bays PM, Husain M (2008). Dynamic shifts of limited working memory resources in human vision. Science (New York, NY).

[R4] Bays PM, Taylor R (2018). A neural model of retrospective attention in visual working memory. Cognitive Psychology.

[R5] Ecker UKH, Lewandowsky S, Oberauer K (2014). Removal of information from working memory: A specific updating process. Journal of Memory and Language.

[R6] Ecker UKH, Oberauer K, Lewandowsky S (2014). Working memory updating involves item-specific removal. Journal of Memory and Language.

[R7] Fawcett JM, Taylor TL (2008). Forgetting is effortful: Evidence from reaction time probes in an item-method directed forgetting task. Memory & Cognition.

[R8] Fougnie D, Suchow JW, Alvarez GA (2012). Variability in the quality of visual working memory. Nature Communications.

[R9] Galletly CA, MacFarlane AC, Clark CR (2007). Impaired updating of working memory in schizophrenia. International Journal of Psychophysiology.

[R10] Griffin IC, Nobre AC (2003). Orienting Attention to Locations in Internal Representations. Journal of Cognitive Neuroscience.

[R11] JASP Team (2020). JASP (Version 0.14.1)[Computer software].

[R12] Kessler Y, Meiran N (2006). All updateable objects in working memory are updated whenever any of them are modified: Evidence from the memory updating paradigm. Journal of Experimental Psychology: Learning, Memory, and Cognition.

[R13] Kessler Y, Meiran N (2008). Two dissociable updating processes in working memory. Journal of Experimental Psychology: Learning, Memory, and Cognition.

[R14] Kessler Y, Rac-Lubashevsky R, Lichtstein C, Markus H, Simchon A, Moscovitch M (2015). Updating visual working memory in the change detection paradigm. Journal of Vision.

[R15] Keysers C, Gazzola V, Wagenmakers E-J (2020). Using Bayes factor hypothesis testing in neuroscience to establish evidence of absence. Nature Neuroscience.

[R16] Kim H, Smolker HR, Smith LL, Banich MT, Lewis-Peacock JA (2020). Changes to information in working memory depend on distinct removal operations. Nature Communications.

[R17] Landman R, Spekreijse H, Lamme VAF (2003). Large capacity storage of integrated objects before change blindness. Vision Research.

[R18] LaRocque JJ, Lewis-Peacock JA, Drysdale AT, Oberauer K, Postle BR (2013). Decoding Attended Information in Short-term Memory: An EEG Study. Journal of Cognitive Neuroscience.

[R19] Levens SM, Gotlib IH (2010). Updating positive and negative stimuli in working memory in depression. Journal of Experimental Psychology: General.

[R20] Lewis-Peacock JA, Drysdale AT, Oberauer K, Postle BR (2011). Neural Evidence for a Distinction between Short-term Memory and the Focus of Attention. Journal of Cognitive Neuroscience.

[R21] Lewis-Peacock JA, Drysdale AT, Oberauer K, Postle BR (2012). Neural Evidence for a Distinction between Short-term Memory and the Focus of Attention. Journal of Cognitive Neuroscience.

[R22] Lewis-Peacock JA, Kessler Y, Oberauer K (2018). The removal of information from working memory: The removal of information from working memory. Annals of the New York Academy of Sciences.

[R23] Liang F, Paulo R, Molina G, Clyde MA, Berger JO (2008). Mixtures of g Priors for Bayesian Variable Selection. Journal of the American Statistical Association.

[R24] Lieder I, Adam V, Frenkel O, Jaffe-Dax S, Sahani M, Ahissar M (2019). Perceptual bias reveals slow-updating in autism and fast-forgetting in dyslexia. Nature Neuroscience.

[R25] Maxcey-Richard AM, Hollingworth A (2013). The strategic retention of task-relevant objects in visual working memory. Journal of experimental psychology Learning, memory, and cognition.

[R26] Meiran N, Diamond GM, Toder D, Nemets B (2011). Cognitive rigidity in unipolar depression and obsessive compulsive disorder: Examination of task switching, Stroop, working memory updating and post-conflict adaptation. Psychiatry Research.

[R27] Moores KA, Clark CR, McFarlane AC, Brown GC, Puce A, Taylor DJ (2008). Abnormal recruitment of working memory updating networks during maintenance of trauma-neutral information in post-traumatic stress disorder. Psychiatry Research: Neuroimaging.

[R28] Oberauer K (2001). Removing irrelevant information from working memory: A cognitive aging study with the modified Sternberg task. Journal of Experimental Psychology: Learning, Memory, and Cognition.

[R29] Oberauer K (2018). Removal of irrelevant information from working memory: sometimes fast, sometimes slow, and sometimes not at all. Annals of the New York Academy of Sciences.

[R30] Oberauer K, Farrell S, Jarrold C, Pasiecznik K, Greaves M (2012). Interference between maintenance and processing in working memory: The effect of item–distractor similarity in complex span. Journal of Experimental Psychology: Learning, Memory, and Cognition.

[R31] Oberauer K, Lin H-Y (2017). An interference model of visual working memory. Psychological Review.

[R32] Palmer J (1990). Attentional limits on the perception and memory of visual information. Journal of Experimental Psychology: Human Perception and Performance.

[R33] Pertzov Y, Bays PM, Joseph S, Husain M (2013). Rapid Forgetting Prevented by Retrospective Attention Cues. Journal of Experimental Psychology: Human Perception and Performance.

[R34] Prinzmetal W, Amiri H, Allen K, Edwards T (1998). Phenomenology of attention: I. Color, location, orientation, and spatial frequency. Journal of Experimental Psychology: Human Perception and Performance.

[R35] Schneegans S, Bays PM (2017). Neural Architecture for Feature Binding in Visual Working Memory. The Journal of Neuroscience.

[R36] Schneegans S, Bays PM (2018). Drift in Neural Population Activity Causes Working Memory to Deteriorate Over Time. The Journal of Neuroscience.

[R37] Schneegans S, Taylor R, Bays PM (2020). Proceedings of the National Academy of Sciences.

[R38] Souza AS, Oberauer K (2016). In search of the focus of attention in working memory: 13 years of the retro-cue effect. Attention, Perception, & Psychophysics.

[R39] Souza AS, Rerko L, Lin H-Y, Oberauer K (2014). Focused attention improves working memory: implications for flexible-resource and discrete-capacity models. Attention, Perception, & Psychophysics.

[R40] Souza AS, Rerko L, Oberauer K (2014). Unloading and reloading working memory: attending to one item frees capacity. Journal of experimental psychology Human perception and performance.

[R41] Stefan AM, Gronau QF, Schönbrodt FD, Wagenmakers E-J (2019). A tutorial on Bayes Factor Design Analysis using an informed prior. Behavior Research Methods.

[R42] Stokes MG (2015). ‘Activity-silent’ working memory in prefrontal cortex: a dynamic coding framework. Trends in Cognitive Sciences.

[R43] Tabi YA, Husain M, Manohar SG (2019). Recall cues interfere with retrieval from visuospatial working memory. British Journal of Psychology.

[R44] Tabi YA, Maio MR, Fallon SJ, Udale R, Dickson S, Idris MI, Husain M (2021). Impact of processing demands at encoding, maintenance and retrieval in visual working memory. Cognition.

[R45] Taylor R, Bays PM (2018). Efficient coding in visual working memory accounts for stimulus-specific variations in recall. The Journal of Neuroscience.

[R46] Taylor R, Bays PM (2020). Theory of neural coding predicts an upper bound on estimates of memory variability. Psychological Review.

[R47] van den Berg R, Shin H, Chou WC, George R, Ma WJ (2012). Variability in encoding precision accounts for visual short-term memory limitations. Proceedings of the National Academy of Sciences.

[R48] van Raalten TR, Ramsey NF, Jansma JM, Jager G, Kahn RS (2008). Automatization and working memory capacity in schizophrenia. Schizophrenia Research.

[R49] Vogel E, Woodman G, Luck S (2006). The time course of consolidation in visual working memory. J Exp Psychol Hum Percept Perform.

[R50] Wagenmakers E-J, Marsman M, Jamil T, Ly A, Verhagen J, Love J, Morey RD (2018). Bayesian inference for psychology. Part I: Theoretical advantages and practical ramifications. Psychonomic Bulletin & Review.

[R51] Weber DL, Clark CR, McFarlane AC, Moores KA, Morris P, Egan GF (2005). Abnormal frontal and parietal activity during working memory updating in post-traumatic stress disorder. Psychiatry Research: Neuroimaging.

[R52] Wilken P, Ma WJ (2004). A detection theory account of change detection. Journal of Vision.

[R53] Williams M, Hong SW, Kang M-S, Carlisle NB, Woodman GF (2013). The benefit of forgetting. Psychonomic Bulletin & Review.

[R54] Williams M, Woodman GF (2012). Directed forgetting and directed remembering in visual working memory. Journal of Experimental Psychology: Learning, Memory, and Cognition.

[R55] Zhang W, Luck SJ (2008). Discrete fixed-resolution representations in visual working memory. Nature.

