# Supplementary material for: Working memory is updated by reallocation of resources from obsolete to new items
Source: Atten Percept Psychophys. 2022 Oct 17;85(5):1437–51. doi: 10.3758/s13414-022-02584-2 (PMC7614821; doi:10.3758/s13414-022-02584-2)
Supplement: Supplementary file 1 — (DOCX 653 kb) [file 13414_2022_2584_MOESM1_ESM.docx]

**Supplementary Materials**

**Experiment 1: Analysis of First Array Probes**

Figure S1. Circular standard deviation for first array probes.

Table S1. Comparison of recall for first array probes.

| **Condition 1** | **Condition 2** | **BF₁₀** |
| --- | --- | --- |
| No update | Repeat | 0.293 |
| No update | Replace | 2.317 |
| No update | New | 0.727 |
| Repeat | Replace | 2.389 |
| Repeat | New | 0.884 |
| Replace | New | 0.480 |
| No update | Replace (corrected) | 1.396 |
| Repeat | Replace (corrected) | 1.014 |
| New | Replace (corrected) | 0.288 |
| *Bayesian paired samples t-test.* | | |

**Experiment 2: Analysis of First Array Probes**

Figure S2. Circular standard deviation for first array probes.

Table S2. Comparison of recall for first array probes.

| **Condition 1** | **Condition 2** | **BF₁₀** |
| --- | --- | --- |
| No update | New | 5.54 x 10^4 |
| *Bayesian paired samples t-test.* | | |

**Experiment 3: Analysis of First Array Probes**

Figure S3. Circular standard deviation for first array probes.

Table S3. Pairwise comparison of recall for first array probes.

| **Condition 1** | **Condition 2** | **BF₁₀** |
| --- | --- | --- |
| 1s | 2s | 0.319 |
| 2s | 4s | 1.111 |
| *Bayesian paired samples t-test.* | | |

**Mixture Model Parameters**

Mixture model was fitted to pooled subject data. Below we provide the best fitting parameter estimates.

Table S4. Global mixture model fits to replace recall data.

| Exp. | Delay | Array |  | SD | p(Mem) | p(NT) | p(Guess) |  | Total | Intru. | N |
| --- | --- | --- | --- | --- | --- | --- | --- | --- | --- | --- | --- |
| 1 | 1s | 1 |  | .40 | .87 | .05 | .08 |  | 20 | 9 | 576 |
|  | 1s | 2 |  | .32 | .94 | .06 | .00 |  | 12 | 10 | 288 |
|  |  |  |  |  |  |  |  |  |  |  |  |
| 2 | 1s | 2 |  | .31 | .84 | .11 | .06 |  | 86 | 52 | 864 |
|  |  |  |  |  |  |  |  |  |  |  |  |
| 3 | 1s | 2 |  | .32 | .87 | .03 | .10 |  | 0 | 0 | 480 |
|  | 2s | 2 |  | .33 | .86 | .10 | .04 |  | 46 | 27 | 480 |
|  | 4s | 2 |  | .29 | .77 | .13 | .10 |  | 65 | 33 | 480 |
